# Monocytes-Derived Macrophages Mediated Stable Expression of Human Brain-Derived Neurotrophic Factor, a Novel Therapeutic Strategy for NeuroAIDS

**DOI:** 10.1371/journal.pone.0082030

**Published:** 2014-02-05

**Authors:** Jing Tong, Shilpa Buch, Honghong Yao, Chengxiang Wu, Hsin-I Tong, Youwei Wang, Yuanan Lu

**Affiliations:** 1 MOE Key Laboratory of Combinatorial Biosynthesis and Drug Discovery, Institute of TCM & Natural Products, School of Pharmaceutical Sciences, Wuhan University, Wuhan, People's Republic of China; 2 Department of Public Health Sciences, University of Hawaii at Manoa, Honolulu, Hawaii, United States of America; 3 University of Nebraska Medical Center, Pharmacology and Experimental Neuroscience, Nebraska Medical Center, Omaha, Nebraska, United States of America; University of Nebraska Medical Center, United States of America

## Abstract

HIV-1 associated dementia remains a significant public health burden. Clinical and experimental research has shown that reduced levels of brain-derived neurotrophic factor (BDNF) may be a risk factor for neurological complications associated with HIV-1 infection. We are actively testing genetically modified macrophages for their possible use as the cell-based gene delivery vehicle for the central nervous system (CNS). It can be an advantage to use the natural homing/migratory properties of monocyte-derived macrophages to deliver potentially neuroprotective BDNF into the CNS, as a non-invasive manner. Lentiviral-mediated gene transfer of human (h)BDNF plasmid was constructed and characterized. Defective lentiviral stocks were generated by transient transfection of 293T cells with lentiviral transfer plasmid together with packaging and envelope plasmids. High titer lentiviral vector stocks were harvested and used to transduce human neuronal cell lines, primary cultures of human peripheral mononocyte-derived macrophages (hMDM) and murine myeloid monocyte-derived macrophages (mMDM). These transduced cells were tested for hBDNF expression, stability, and neuroprotective activity. The GenomeLab GeXP Genetic Analysis System was used to evaluate transduced cells for any adverse effects by assessing gene profiles of 24 reference genes. High titer vectors were prepared for efficient transduction of neuronal cell lines, hMDM, and mMDM. Stable secretion of high levels of hBDNF was detected in supernatants of transduced cells using western blot and ELISA. The conditioned media containing hBDNF were shown to be protective to neuronal and monocytic cell lines from TNF-α and HIV-1 Tat mediated cytotoxicity. Lentiviral vector-mediated gene transduction of hMDM and mMDM resulted in high-level, stable expression of the neuroprotective factorBDNF *in vitro*. These findings form the basis for future research on the potential use of BDNF as a novel therapy for neuroAIDS.

## Introduction

NeuroAIDS is one of the most devastating consequences of HIV-1 infection which incorporates both infectious and degenerative pathophysiologic pathways. Symptoms include behavioral abnormalities, motor dysfunction, and frank dementia [1]. Clinical severity ranges from asymptomatic neurocognitive impairment (ANI) and mild neurocognitive disorder (MND) to its most severe form - HIV-associated dementia (HAD) [2]. Neurologic disorders associated with HIV-1 infection occur in about 40% to 70% of HIV-infected individuals [3]. Despite the improved life expectancy of HIV-1-infected patients due to the advent of highly active antiretroviral therapy (HAART there is an increased prevalence of neurocognitive impairment infected individuals [4]–[7]. Part of this increase may be attributed to poor penetration of HAART drugs into the CNS to eradicate the virus [8] and increased life expectancy of HIV-1-infected patients. Therefore, the development of HAD is a significant independent risk factor in AIDS-related mortality [9].

The blood brain barrier (BBB) strictly limits the transport of soluble materials, pathogens and circulating cells into the brain and helps maintain the stability of the neuronal microenvironment within the CNS. Among the many FDA approved anti-HIV/AIDS drugs, Zidovudine is the only drug known to cross the BBB to some extent [10]. In addition, emergence of distinct drug resistant viral strains has been reported in both plasma and cerebrospinal fluid (CSF) [11]. Therefore, development of adjunctive therapeutic interventions for neuroAIDS is needed.

Neurotrophins are critical secreted peptides that are involved in the differentiation, growth, and survival of neuronal cell populations [12]–[14]. Human BDNF which is abundant and widely expressed in the CNS [15]–[17], is the most commonly studied neurotrophic factor associated with HIV-1 related neuronal injury [18].Animal model in vivo study has shown that BDNF is a powerful neuroprotective agent for degenerating neurons in HAD [19]. Clinically, it has been shown that expression of hBDNF is down-regulated in HIV-positive individuals when compared to HIV-negative individuals [20]. Despite several reports describing the important role of hBDNF in reducing HIV-1 protein-mediated neurotoxicity [21]–[24], inefficient delivery has hampered present attempts to develop hBDNF-based therapies. Moreover, the characteristics of HAD requires continuous and sustained, long-term replenishment of hBDNF in order to alleviate ongoing neuroinflammation.

Neuroinflammation is a common feature observed throughout the progression of the disease from the latent, asymptomatic stage of AIDS to HAD [25]. Circulating monocytes and macrophages have the ability to traverse the intact BBB and undergo differentiation within brain parenchyma, giving rise to long-lived brain resident macrophages and microglia [26]. Furthermore, neuroinvasion of monocytes has been shown to occur concurrently with the detection of virus in the CNS as well as CSF [27]. This is also accompanied by elicitation of chemokines such as MCP-1 by resident CNS cells including neurons, astrocytes and microglia. Chemokines also have the potential to recruit peripheral leukocytes across the BBB into the brain parenchyma [28]. This property of monocytes can be exploited to deliver exogenous genes of interest into the brain for neurotherapy [29]. For example, Dou *et al.* used macrophages as carriers to deliver nanoformulated antiretroviral drugs across the BBB into the various regions of the diseased brain [30]. Previous results from our laboratory showed that intravenously infused primary mouse monocytes were able to transmigrate across intact BBB into the brain, and that we could enhance this process significantly by transient disruption of the BBB [31]. Therefore, the development of a monocyte-/macrophage-based expression of hBDNF could be a harnessed as a possible gene therapy for neuroAIDS.

HIV-based defective lentiviral vectors (LVs) were chosen to evaluate the efficacy of genetically modified MDMs to deliver hBDNF into the CNS due to their ability to transduce dividing and nondividing cells and previously reported marked superiority to other viral vector systems [32]. LVs have the unique ability to deliver relatively large genes or multiple gene inserts, thereby providing controllable and cell-specific expression of the transgene [33]. An early phase clinical trial using LVs as a method for delivery of transgenesfor treatment of CNS disease is currently underway in France [34].

In this study, we constructed an HIV-1-based vector that constitutively expresses hBDNF under the human cytomegalovirus (CMV) promoter, which stably transduced both human and murine monocyte-derived macrophages with high efficiency *in vitro*. The secreted hBDNF protein was shown to be protective for neuronal and macrophagic cell lines following exposure to the neurotoxin TNF-α. Potential adverse effects due to the introduction and expression of hBDNF on transduced cells were evaluated using a GeXP multiplexed analyzer by comparatively assessing the expression of 24 house- keeping genes. Our findings establish the groundwork for hBDNF as a potential novel therapeutic strategy for neuroAIDS through MDM-mediated delivery.

## Results

### Characterization of gene transfer efficiency and stable expression of hBDNF in human neuronal cell lines

Given their ability to transduce non-dividing cells, LVs are an ideal gene delivery vehicle for the transduction of primary cells [35]. This study demonstrated the feasibility of using LVs to introduce hBDNF gene into MDMs. LV-mediated gene transfer efficiency was evaluated initially in human neuronal cell lines. The stable expression of the transgene and its potential adverse impact on the transduced cells were also monitored.

**Figure 1:**
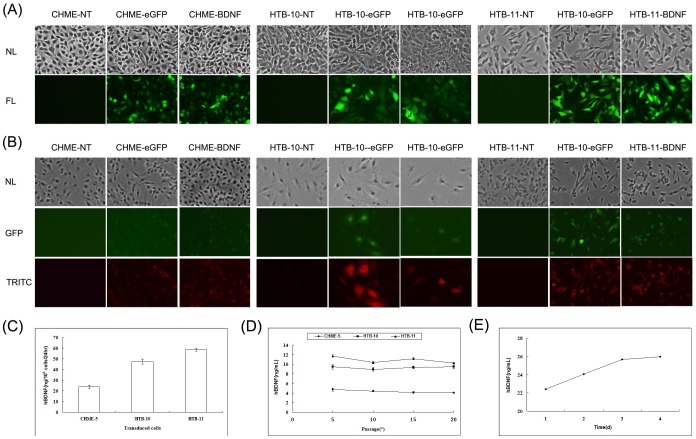
LV-transduced neuronal cell lines. 5×10^5^ HTB-10, HTB-11 and CHME-5 cells were subcultured in 25 cm^2^ tissue culture flask 24 hrs before transduction, then removed the media, DPBS washed cells twice, added 0.5 mL vector suspension (1×10^7^ IU/mL, MOI = 10, for HTB-11, MOI = 1) containing 8 µg/mL polybrene, and incubated at 37°C for 2 hours with gentle mixing every 15 minutes. Then aspirated vector suspension and added 4 mL of fresh growth medium and incubated at 37°C with 5% CO_2_. The medium was replaced 24 hours post-infection and observed cells on day 3 post-infection under fluorescence microscope (Nikon Eclipse TE2000-U). (A) Photomicrographs of LV-transduced neuronal cell lines showing GFP exression. NL: Normal Light. FL: Fluorescent Light. (B) Immunofluorescent staining of GFP in neuronal cell lines. NL: Normal Light. GFP: overlay of green fluorescence. TRITC: overlay of red fluorescence. (C) Moderate to high levels of secreted hBDNF in LV-transduced neuronal cells conditioned media quantified by ELISA. Capture and detection antibodies were rabbit anti-human BDNF, goat anti-human IgG-Biotin respectively. (D) Stable expression of hBDNF detected from transduced neuronal cells. ELISA was performed every 5 passages for 20 passages to assess long-term stable expression of hBDNF gene. (E) Accumulative expression of hBDNF detected in LV-transduced HTB-11 cells.

Human microglial cell line CHME-5, neuroepithelioma cell line HTB-10 and neuroblastoma cell line HTB-11 were transduced with LVs expressing hBDNF under a CMV promoter, and linked to an enhanced green fluorescent protein (eGFP) via the IRES element. Transduction efficiencies were evaluated at day 3 post-transduction by counting the number of GFP-positive cells using a fluorescence microscope. The transduction efficiencies ranged from 98% to 100% among these three neuronal cell lines (Figure 1). This was confirmed by examining the transduced cells for the eGFP expression using an immunofluorescence assay with anti-GFP specific antibody (Figure 1B). Our findings suggest that expression of the genes co-expressed through an IRES element was weaker than the promoter-proximal gene(s) [36].

Expression and secretion of hBDNF from transduced cell lines were determined by western blot analysis. To assess hBDNF protein production extracellularly and intracellularly, culture supernatants and cell lysates from LV-hBDNF- and LV-eGFP-transduced cells (mock cells), and non-transduced cells were collected and extracted. As expected, there was no detection of hBDNF expression in the supernatant from non-transduced and mock cells, whereas hBDNF was detected in both the supernatants and cytosolic fraction of cell lysates from LV-hBDNF-transduced cells. The molecular weight of secreted and intracellular form of hBDNF was approximately 14 kD (data not shown).

We also used a cellular model to test whether the transgene was able to drive the production of a functional hBDNF in cultured supernatants of transduced cells. Transduced cells were seeded at a density of 1.0×10^6^ cell/T-25 cm^2^ flask and cultured at 37°C with 5% CO_2_ for 24 hours. The supernatants were collected and the hBDNF protein concentration was quantified by ELISA. ELISA quantification showed no evidence of hBDNF production in the conditioned media from non-transduced or LV-eGFP-transduced cells. For LV-hBDNF-transduced CHME-5 cells, the hBDNF concentration was determined to be 23.635±1.56 ng/10^6^ cells/24 h. The hBDNF level in the supernatant from LV-hBDNF-transduced HTB-10 was 47.28±2.26 ng/10^6^ cells/24 h. hBDNF levels in the supernatants from LV-hBDNF-transduced HTB-11 cells were the highest, at 58.64±1.34 ng/10^6^ cells/24 hours after just a single transduction event (Figure 1C).

To determine the stability of hBDNF expression and secretion, transduced cells were sub-cultured *in vitro* up to 20 times, and the concentrations of hBDNF in conditioned media was assessed by ELISA quantification at every 5^th^ passage. The level of hBDNF expression was stable over the course of 20 passages (Figure 1D) in all the LV-hBDNF transduced cells (CHME-5, HTB-10 and HTB-11). In addition, we also demonstrated the accumulation of hBDNF in LV-hBDNF transduced HTB-11 cells during a four-day examination (Figure 1E). These results suggest LVs are able to mediate an effective gene transfer into human neuronal cells with high level of stable hBDNF expression.

### Potential adverse impact

**Figure 2:**
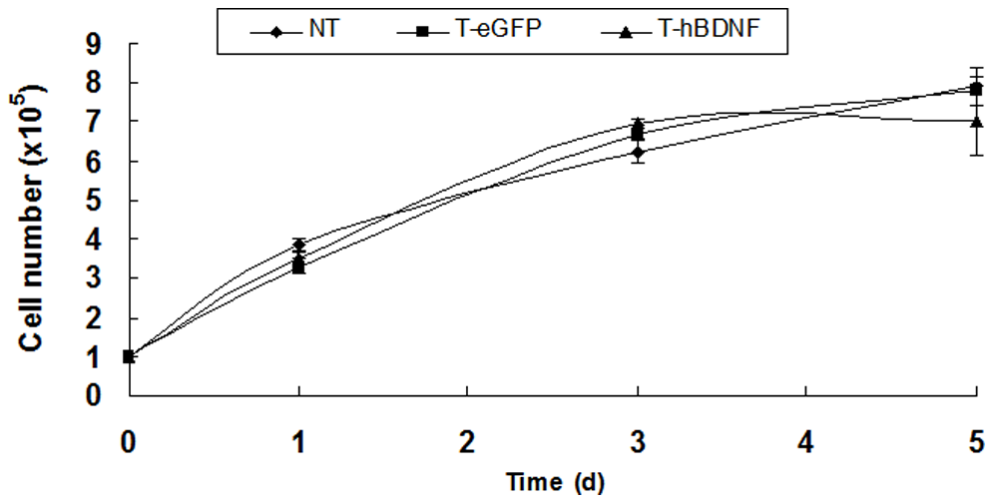
Comparative analysis of the growth kinetics of LV-transduced HTB-11 cells by MTT assay. HTB-11 cells were seeded in 48-well plate at 1×10^5^ cells/mL, then cultured at 37°C, counted cells at day 1, 3, 5. No significant difference was detected. The error bars denote the SD from four independent experimental tests. NT: Non-transduced cells; T-hBDNF: LV-hBDNF transduced HTB-11; T-eGFP: LV- eGFP transduced HTB-11.

The hBDNF gene is the member of the neurotrophin family known to cause distinct widespread trophic effects on neurons both in the peripheral nervous system and CNS [14]. Thus, we conducted tests to evaluate cell growth and kinetics of the transduced neuronal cells. As shown in Figure 2, comparative analysis of cellular morphology and growth kinetics showed no apparent differences between the LV-hBDNF-transduced and non-transduced HTB-11 cells.

### Protection of transduced neuronal cell lines from cytokine/viral protein-mediated neurotoxicity

**Figure 3:**
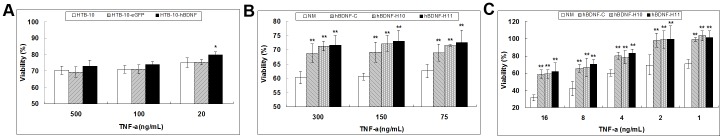
hBDNF mediated protection from TNF-α. (A) LV-hBDNF-transduced, LV-eGFP-transduced, and untransduced normal HTB-10 were treated for 2 days at 37°C with TNF-α at three selected concentrations ranging from 20 to 500 ng/mL, and their viability was determined by MTT assay. The cell viability of normal cells without TNF-α treatment was normalized as 100%. Transduced cells expressing hBDNF showed significantly higher viability than the other two control HTB-10 cells at concentration 20 ng/mL of TNF-α (^*^ *p*<0.05). Results shown represent mean levels of four independent experiments and error bars denote the standard deviation. (B) Normal HTB-10 cells were exposed to selected concentrations of TNF-α (75 to 300 ng/mL) in the absence or presence of cell culture media (1∶10 dilution) collected from LV-hBDNF-transduced neuronal cells. Following incubation at 37°C for 2 days, cell viability was determined by MTT assay. The viability of cells without TNF-α treatment was defined as 100%. Viability of test cells was significantly higher in the presence of conditioned media from transduced cells expressing hBDNF (^**^ *p*<0.01). NM: normal medium; hBDNF-C: condition medium from LV-hBDNF-transduced CHME-5; hBDNF-H10: condition medium from LV-hBDNF-transduced HTB-10. hBDNF-H11: condition medium from LV-hBDNF-transduced HTB-11. Results shown represent mean levels of four independent experiments and error bars denote the standard deviation. (C) Human monocytic U937 cells were treated with selected concentrations of TNF-α (2–16 ng/mL) in the presence or absence of cell culture media (1∶10 dilution) from LV-hBDNF-transduced cells as described in (B). Significant protection was detected for the test cells when the conditional medium from the transduced cells was present (*p*<0.01).

**Figure 4:**
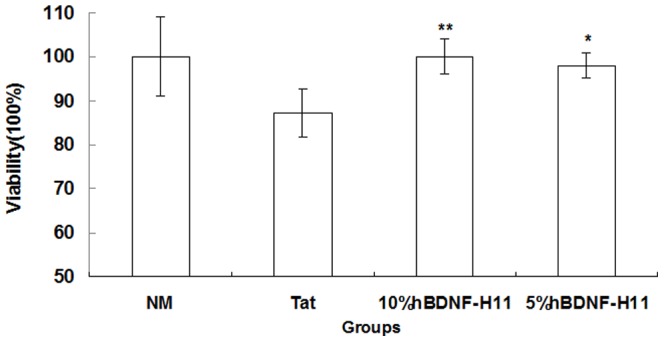
hBDNF mediated protection from Tat to transduced cells. SH-SY5Y cells were pretreated with conditioned media (1∶10 & 1∶20) from LV-hBDNF-transduced HTB-11 cells for 1 hour, then exposed to HIV-1 Tat protein (200 ng/mL) for 48 hours, and cell viability was determined by MTT assay. The viability of cells without Tat treatment was defined as 100%. Viability of test cells was significantly higher in the presence of conditioned media from transduced HTB-11 cells expressing hBDNF (^**^ *p*<0.01; ^*^ *p*<0.05). Results shown represent mean levels of three independent experiments and error bars denote the standard deviation. NM: normal medium; Tat: treated with Tat at concentration of 200 ng/mL without condition medium from LV-hBDNF-transduced HTB-11; 10%hBDNF-H11: 1∶10 diluted condition medium from LV-hBDNF-transduced HTB-11; 5%hBDNF-H11: 1∶20 diluted condition medium from LV-hBDNF-transduced HTB-11.

We next wanted to determine whether the expression of hBDNF would provide neuroprotection against HIV-1 protein and TNF-α cytotoxicity. TNF-α is an important mediator of inflammation in HAD. Increased levels of TNF-α in the CNS of patients with HAD has largely been attributed to the exposure of brain macrophages and microglia to HIV-1 proteins including HIV-1 Tat [37]. In fact, TNF-α is the major contributor to HIV-1 Tat mediated neurotoxicity [38]–. Following exposure to different concentrations of TNF-α, LV-hBDNF-, LV-eGFP-, and non-transduced HTB-10 cells were comparatively evaluated by examining cell viability using the MTT assay. Intriguingly, cells expressing hBDNF demonstrated increased viability compared with mock or non-transduced HTB-10 cells at a concentration of 20 ng/mL (*p*<0.05) (Figure 3A) in response to TNF-α. To examine if conditioned medium containing hBDNF could also offer protection, HTB-10 and U937 cells were exposed to different concentrations of TNF-α in the presence or absence of the conditioned media from LV-hBDNF-transduced cells (CHME-5, HTB-10, and HTB-11). Conditioned media from LV-hBDNF transduced cells provided significant protection to HTB-10 and U937 cells against TNF-α-mediated toxicity (*p*<0.01) (Figure 3B and 3C). However, media collected from mock or non-transduced control cells (CHME-5, HTB-10 and HTB-11) failed to show any detectable protection against TNF-α-mediated toxicity (Figure not shown). Furthermore, conditioned media from LV-hBDNF-transduced HTB-11 cells in different dilutions were also protective to human neuroblastoma SH-SY5Y cells exposed to HIV-1 Tat at concentration of 200 ng/mL (*p*<0.01 and *p*<0.05) (Figure 4).

### Characterization of gene transfer efficiency and expression of hBDNF in human/murine monocyte-derived macrophages

**Figure 5:**
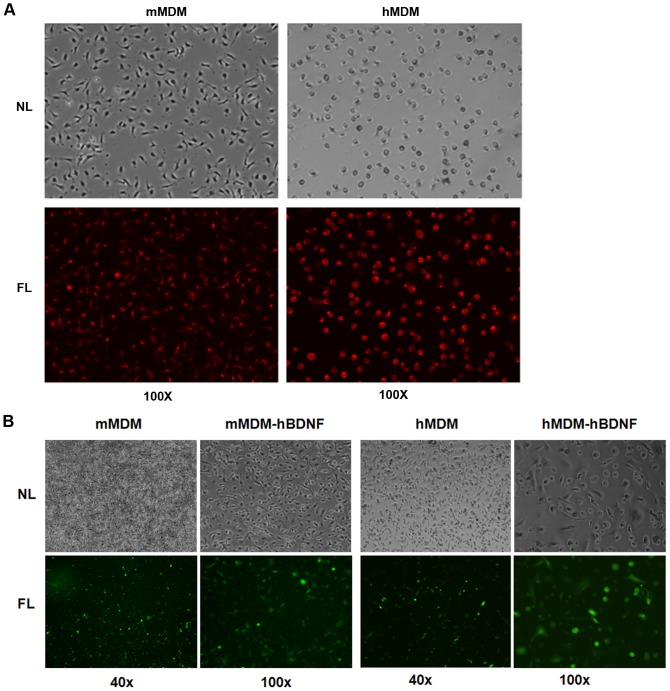
LV-hBDNF mediated transduction of primary MDM cultures. (A) Photomicrographs of primary cultures of hMDM and mMDM at day 6 stained with selected PE-conjugated antibodies (CD14 for hMDM and CD11b for mMDM). (B) Photomicrographs of primary cultures of hMDM and mMDM transduced with LV-BDNF vector (MOI = 50) at day 6 cultivation time in the presence of polybrene (8 mg/mL Polybrene). The transduced cells showed GFP expression at day 6 post-transduction time using inverted light and fluorescence microscopy.

Human monocyte-derived macrophages were isolated according to the method described previously [40]. Purity of hMDMs (greater than 98%) was confirmed by immunocytochemistry (ICC) staining with PE-conjugated antibodies against human CD14, a cell surface marker for monocytes and macrophages (Figure 5A). These cells were transduced with LVs expressing hBDNF and eGFP at day 6 post-isolation and the transduction efficiencies were evaluated at day 6 post-transduction by counting the number of GFP-positive cells with a randomly selected cell population using a fluorescence microscope. The transduction efficiencies were determined to be over 50% (Figure 5B). To evaluate the migratory ability of transduced MDM and subsequent therapeutic potential, primary cultures of mouse MDMs (mMDM) were prepared according to the method described previously [32]. Purity of the primary cultures of mMDM was tested and confirmed using PE-conjugated antibody against mouse CD11b (Figure 5A). These cells were transduced with LVs expressing hBDNF and eGFP at day 4 post-isolation, the transduction efficiencies were evaluated at day 6 post-transduction by counting the number of GFP-positive cells using a fluorescence microscope. The transduction efficiencies for mMDMDs were estimated to be as high as 80% (Figure 5B).

**Figure 6:**
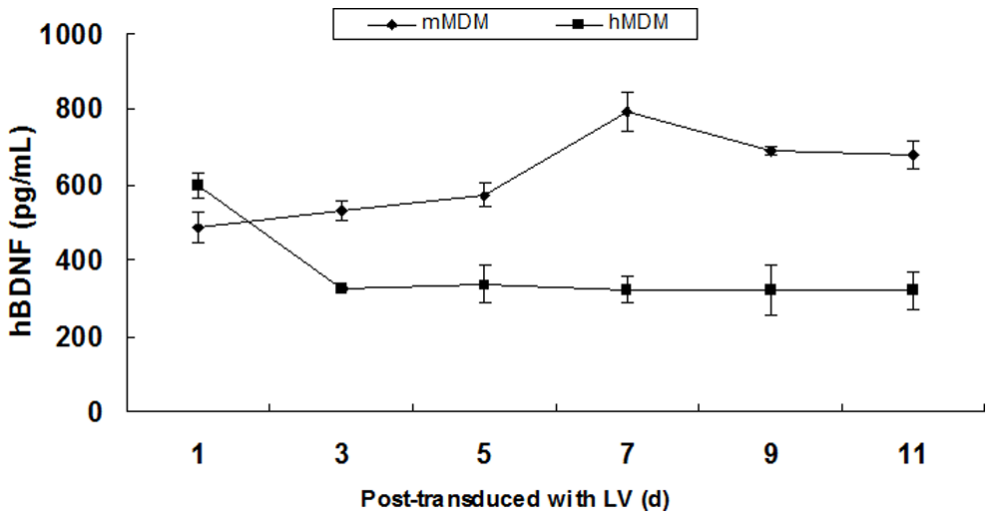
Stable expression of hBDNF in LV-transduced primary MDM quantified by ELISA. ELISA was performed every 2 days to assess long-term stable expression of hBDNF gene in hMDM and mMDM.

Expression of hBDNF from the transduced primary cultures of MDM was quantified by ELISA. The hBDNF level in the supernatant from LV-hBDNF-transduced hMDM was over 300 pg/mL. It was shown that the level of hBDNF expression in the supernatants of LV-hBDNF-transduced mMDM reachedover 500 pg/mL. The expression of hBDNF in transduced cells remained stable up to day 11 post-transduction (Figure 6). Morphology of the transduced cells showed no difference compared to the non-transduced control cells (data not shown).

### GeXP multiplexed assay

**Figure 7:**
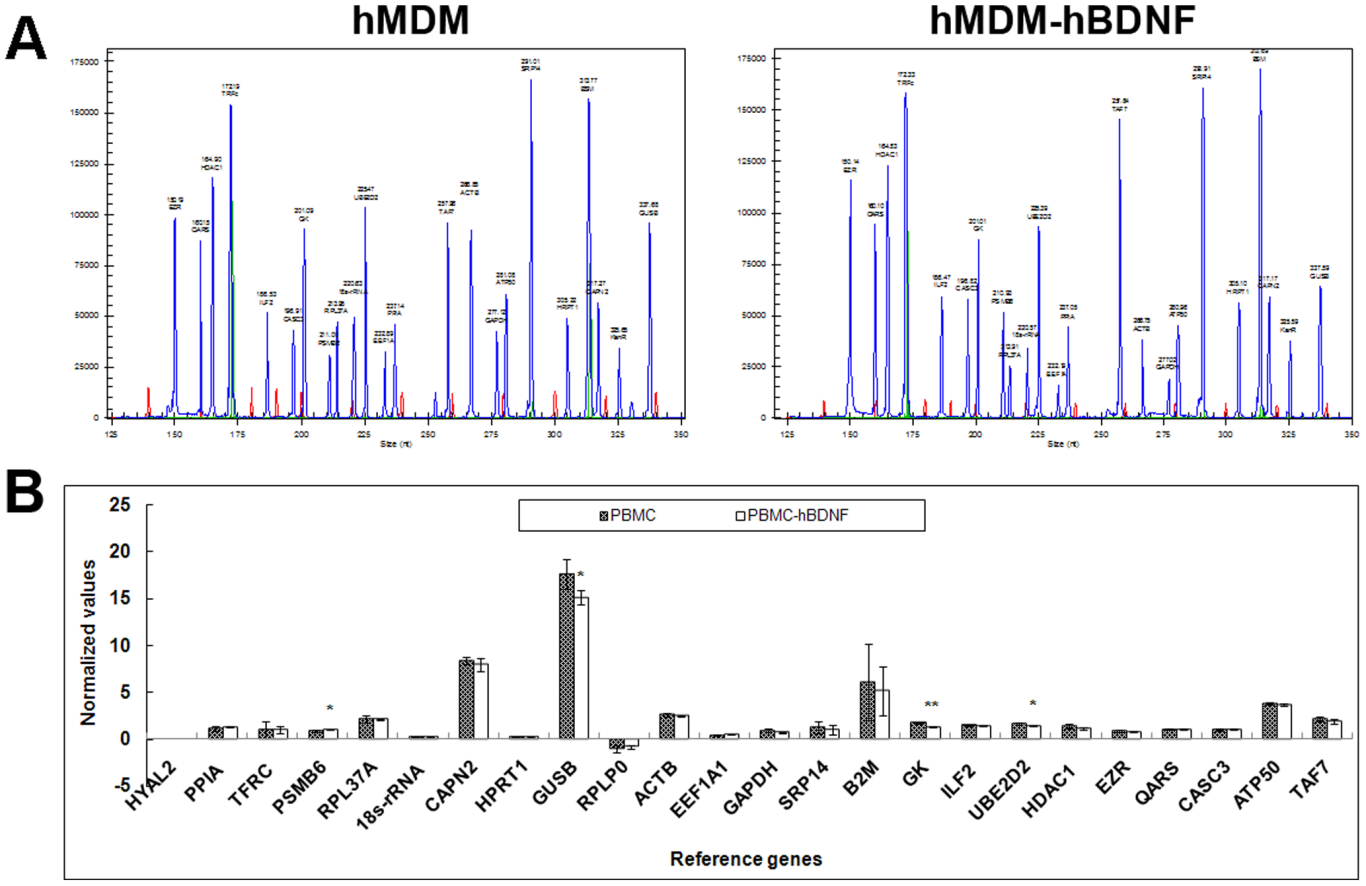
Multiplex primer RT-PCR capillary gel electrophoresis analysis of 24 reference genes of primary hMDM cultures. Total RNA was extracted from both non-transduced cells and LV-transduced cells, and amplified by RP-PCR. Kan^R^ RNA was added to each sample as an internal control and two genes were assigned as reference genes. Finally, amplicons were combined with DNA size standard-400 and sample loading solution and subjected to capillary electrophoresis. Results analyzed by Express Profiler and Quant Tool, were normalized to the internal control Kan^R^ to eliminate any inter-capillary differences and then normalized a second time to the pre-selected reference genes. Lastly, results were compared to the standard curve created for the specific multiplex and normalized values and significance differences between samples were calculated using T-tests. (A) The capillary gel electrophoresis results of hMDM were normalized with HRPT1 and ATP50. (B) Average change of normalized value (±SD) in expression levels following transduction of the 24 genes used in the GeXP assay. (n = 4).

A GenomeLab GeXP Genetic Analysis System (Beckman Coulter, Brea, CA) was employed to comparatively evaluate the expression of 24 basic house- keeping genes (Table 1) between transduced and non-transduced normal cells. Kan^R^ spike was used for intercapillary normalization followed by normalization with HRPT1 and ATP50, and 23 Human Reference Multiplex genes were detected from the primary cultures of hMDM except HYAL2. As shown in Figure 7, all 23 detectable reference genes retained their expression at the same level compared with the non-transduced control hMDM, except for three genes which were down-regulated in LV-hBDNF-tranduced cells [GUSB (15.153±1.568, *p*<0.05), GK (1.295±0.045, *p*<0.01), and UBE2D2 (1.410±0.118, *p*<0.05)] and one gene that was up-regulated (PSMB6 1.004±0.072, *p*<0.05).

**Table 1:** Human Reference Plex Gene Information.

	*Abbr.*	*Reference gene*	*ID*	*Function*	*Size*
1	EZR	Ezrin	X51521	Ezrin-cell surface structure, adhesion, migration, and organization; implicated in various human cancers	150
2	QARS	QRSHs glutaminyl-tRNA synthetase	X76013	QRSHs glutaminyl-tRNA synthetase-catalyzes aminoacylation of tRNA	160
3	HDAC1	Histone deacetylase HD1	U50079	Histone deacetylase HD1-regulation of eukaryotic gene expression	165
4	TFRC	Transferrin receptor	BC001188	Transferrin receptor-importing iron into the cell	172
5	ILF2	Nuclear factoNF45	U10323	Nuclear factor 45-transcription factor required for expression of IL-2 gene	186
6	CASC3	MLN51	X80199	MLN51-Functions in nonsense-mediatedmRNA decay	197
7	GK	glycerol kinase	NM_203391	Glycerol kinase-regulation of glycerol uptake and metabolism	201
8	PSMB6	Proteasome subunit Y	D29012	Proteasome subunit Y -cleaves peptides in an ATP/ubiquitin-dependent process in a non-lysosomal pathway	211
9	RPL37A	Ribosomal protein L37a (RPL37A)	L06499	Ribosomal proteinL37a-catalyzes protein synthesis	214
10	18s-rRNA	18s-rRNA	M10098	Component of eukaryotic small ribosomal subunit	220
11	UBE2D2	E2 UCE UbcH5B	U39317	E2 ubiquitin conjugating enzyme-ubiquitination of tumor-suppressor protein p53	225
12	EEF1A1	Elongation factor EF-1-alpha	NM_001402	Elongation factor alpha-enzymatic delivery of aminoacyl tRNAs to the ribosome	233
13	PPIA	cyclophilin A	BC000689	Cyclophilin A-Cyclosporin binding-protein; role in cyclosporine A-mediated immunosuppression	237
14	HYAL2	Lysosomal hyaluronidase	AJ000099	Lysosomal hyaluronidase-GPI-anchored cell surface protein	253
15	TAF7	Transfer factor IID	X97999	Transcription factor-TATA box binding protein; required for transcription	258
16	ACTB	beta-actin	NM_001101	Beta-actin-involved in cell mobility, structure, and integrity	267
17	GAPDH	GAPDH	NM_002046	Catalyzes an important energy-yielding step in carbohydrate metabolism	277
18	ATP50	ATP synthase	X83218	ATP synthase-involved in transmission of conformational changes	281
19	SRP14	18 kDa Alu RNA BP (SRP14)	NM_003134	18 kDa Alu RNA binding protein-signal recognition particle	291
20	HPRT1	Hypoxanthaine ribosyl transferase	M31642.1	Hypoxanthaine ribosyl transferase-generation of purine nucleotides through purine salvage pathway	305
21	B2M	Beta 2 microglobulin	NM_004048	Beta 2 microglobulin-serum protein found on the surface of nearly all nucleated cells	314
22	CAPN2	Ca2-activated neutral protease large subunit	M23254	Calpain 2-Large subunit of the ubiquitous enzyme	317
23	KANR	Kanamycin resistance*	n/a	Kanamycin resistance	325
24	RPLP0	Acidic Ribosomal Protein (RPLP0)	NM_001002	Acidic ribosomal protein	330
25	GUSB	beta-glucuronidase	NM_000181	Beta-glucuronidase-degrades glycosaminoglycans	338

(*: KANR was used as a positive control spike).

doi:10.1371/journal.pone.0082030.t001

### Protection of transduced MDMs against cytokine/viral protein-mediated toxicity

**Figure 8:**
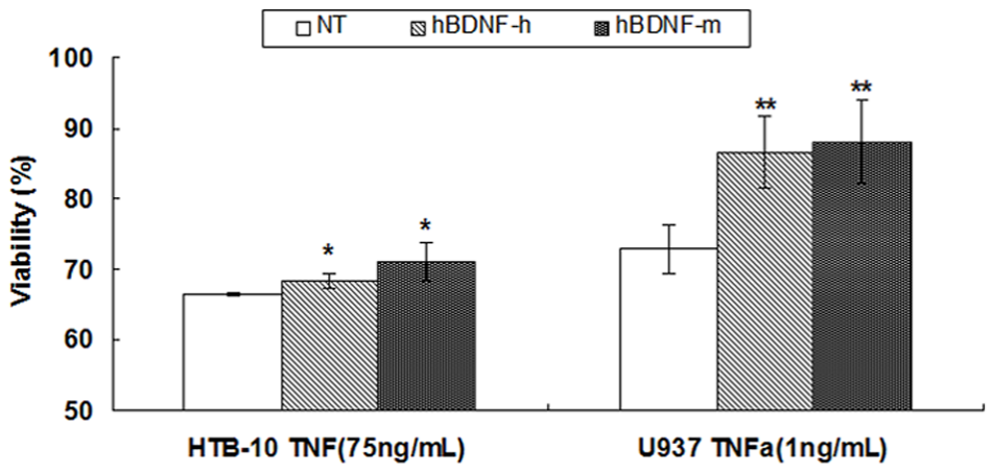
hBDNF secreted from primary MDM cultures transduced by LV-hBDNF vector are neuroprotective against TNF-α. Human HTB-10 and U937 cells were exposed to TNF-α in the presence or absence of conditional media from transduced primary MDM and viability of the test cells were analyzed by MTT as described for figure 3B. Viability was significantly higher for the cells treated with conditioned media from transduced cells expressing hBDNF (^*^ *p*<0.05 in HTB-10; ^**^ *p*<0.01 in U937) compared to the cells receiving TNF-α alone. hBDNF-h: condition medium from LV-hBDNF-transduced hMDM; hBDNF-m: condition medium from LV-hBDNF-transduced mMDM. Results shown represent mean levels of four independent experiments and error bars denote the standard deviation.

HTB-10 and U937 cells were exposed to various concentrations of TNF-α in the presence or absence of conditioned media from primary cultures of transduced hMDM and mMDM. Cell viability assessed by MTT assay demonstrated that media from hBDNF-transduced cells provided significant protection to the human neuronal (*p*<0.05) and monocytic (*p*<0.01) cell lines against TNF-α-mediated toxicity (Figure 8).

## Discussion

Chronic HIV-1 infection can lead to neuroAIDS, a disease comprising both infectious and degenerative pathophysiologic pathways. The pathogenic mechanism of neuroAIDS remains poorly understood, although there is general agreement that the entry of HIV-1 into the CNS occurs shortly after viral infection via infected monocytes or macrophages [41],[42]. These infected peripheral monocytes penetrate into the CNS and pass the virus to microglia and the resident macrophages but not neurons [43],[44]. Infection of macrophages and microglia can lead to cell activation and production of neurotoxic cytokines including TNF-α and the viral proteins includinHIV-1 Tat [45], which can directly or indirectly lead to neuronal damage and apoptosis. Thus, one of the effective therapeutic approaches to combat neuroAIDS is to use neuroprotective effectors to alleviate the neurotoxicity mediated from the viral proteins and cytokines.

Human BDNF belongs to the neurotrophin family of growth factors, which includes nerve growth factor (NGF), neurotrophin-3 (NT-3) and NT4/5. Several independent investigations have suggested that BDNF may help prevent neurotoxic effects of HIV-1 proteins *in vitro* and *in vivo* 21,[46]–[49]. Recent studies have shown that viral proteins can affect the level of BDNF [50],[51], and where serum concentrations of hBDNF in HIV-positive patients was lower than in HIV-negative individuals [20]. A strong correlation was also noted between the severity of neurological symptoms in HIV-positive patients and declining levels of hBDNF [52]. Therefore, hBDNF expressed from transduced cells may have a therapeutic role in reducing or neutralizing neurotoxic effect of both TNF-α and HIV-1 Tat. However, current attempts for neuroAIDS therapy have been largely constrained by two major factors: inability of drugs such as BDNF to cross the BBB to enter the brain parenchyma, and the lack of long-term sustainability of adequate amounts of therapeutic drugs in the brain. Invasive surgery has proven to be an approach to overcome the BBB restriction, but it is often accompanied with traumatic brain injuries and transient availability of uncontrolled amounts of therapeutic drugs [53]. Thus, invasive injection is not a useful therapeutic method since neuroAIDS is a chronic disease, which requires therapeutic treatment to be sustained for long-term in order to protect neurons from continuous onslaught by viral and cellular toxins.

The goal of this study was to test and evaluate the possible use of genetically modified MDM as a noninvasive gene vehicle to deliver hBDNF in to the CNS to treat neuroAIDS. We successfully constructed LVs which hold the promise of transducing primary cultures of MDM prepared from both human whole blood and murine bone marrow [32],[40]. LV-mediated effective introduction of hBDNF into primary cultures of MDM and subsequently stable expression will offer continuous and sustained levels of hBDNF for an extended period of time, an important prerequisite for treatment of a chronic neurodegenerative condition. In the current study, we demonstrated that hBDNF expressed from LV-transduced cells conferred significant neuroprotection (p<0.01) against a pro-inflammatory cytokine TNF-α in several human neuronal cell cultures including neuroblastoma HTB-10 and monocytic U937 cells. This protective effect of BDNF was also evidenced in bone marrow-derived SH-SY5Y neuroblastoma cells exposed to the neurotoxin HIV-1 Tat. These findings suggest that effective delivery of genetically modified primary MDM may lead to long-term constitutive expression of a moderate amount of hBDNF in the brain. This could be an effective therapeutic strategy against neuroAIDS.

A monocyte-/macrophage-based gene delivery system represents a promising therapeutic strategy to attenuate neuroinflammatory processeslinked to neuronal death. Immunocytes including mononuclear phagocytes (dendritic cells, monocytes and macrophages), neutrophils, and lymphocytes are highly mobile and able to transmigrate across impermeable barriers to sites of infection, tissue injury, cancer, or inflammatory disease [54]. There is of a growing interest in testing and establishing the use of mononuclear phagocytic lineage cells, including monocytes and macrophages, as “Trojan horses” for delivery of anti-inflammatory and anti-infective medications into the CNS [55]. Biju *et al.* tested the use of genetically modified monocytes/macrophages to deliver GDNF as a therapy against Parkinson's disease in animal models, demonstrating the role of macrophages as a powerful tool for delivery and expression of therapeutic transgenes at the site(s) of neurodegeneration [56]. In recent clinical gene therapy trials, LV-based gene delivery approaches have successfully been used for genetic modification of hematopoietic stem cells with a corrective gene encoding ABCD1 gene to treat X-linked adrenoleukodystrophy [57]. These results have led toward the development of an MDM cell-based gene therapy approach with the potential for systematic and sustained expression of neuroprotective hBDNF.

GeXP Genetic Analysis is an assay used to detect or diagnose virus types/subtypes, identify the diagnostic biomarkers of diseases, or reveal the relationship between gene expression and disease severity [58]–[60]. We employed GeXP to assess whether LV-mediated gene transduction could result in alteration of gene profiles in the transduced cells. Our findings revealed that while all of these reference genes remained stable, changed gene profiles were detected from transduced hMDM cultures, including three down-regulated genes (GK, GUSB, and UBE2D2) and an up-regulated PSMB6 gene. Among these genes, glycerol kinase (GK) is a phosphotransferase enzyme responsible for regulation of glycerol uptake and metabolism [61]. Human beta-glucuronidase (GUSB) is a member of the glycosidase family 2 with catalytic activity for –D-glucuronic acid residues. E2 ubiquitin conjugating enzyme UbcH5B (UBE2D2) functions in the ubiquitination of the tumor-suppressor protein p53. PSMB6 is the gene that encodes for human proteasome subunit beta type-6, which is expressed in eukaryotic cells and known to cleave peptides in an ATP/ubiquitin-dependent process in the non-lysosomal pathway. Despite the detected change of the genes, transduced hMDM showed no apparent difference as compared to non-transduced cells in terms of morphology and stable growth under the same in vitro conditions (>3 weeks). This may suggest that statistical difference in their expression of these four genes may not indicatea significant alteration of cellular function. A recent study of long-term examination of LV-transduced CHME-5 and HTB-10 cells has revealed no apparent functional alteration of transductant cells including cell morphology and growth kinetics, as well as transgene expression (>20 passages) [62]. Future of transduced MDM specifically at the altered genes will be conducted to ascertain whether LV-mediated transduction and expression of hBDNF may cause any adverse effects or functional changes to transduced cells in order to facilitate the development of MDM-mediated gene therapy for neuroAIDS.

## Experimental Methods

### Ethics Statement

1. Blood was anonymously collected from unidentified health donors with approval from the University of Hawaii Committee on Human Studies (UHCHS); Healthy blood donors signed a written consent form outlining the procedure for this study; both the consent form and procedures were approved by the UHCHS. 2. Laboratory mice were used for experimental tests with approval from the University of Hawaii Animal Care and Use Committee in accordance with the Animal Welfare Act and NIH guidelines.

### Plasmid Construction—Lentiviral Vector Production and Virus Titration

Generation of a HIV-1-based lentiviral vector containing an expression cassette for hBDNF was constructed. Briefly, the synthetic gene was amplified by PCR, using primer pair (5′-GCCTCGAGCTAATGACCATCCTTTTCCTTAC TA-3′ and 5′-GCGGATCCGACCTATCTTCCCCTTTTAATGGT-3′) containing Xho I and BamH I restriction sites within the 5′ and 3′ termini, respectively, and inserted into the pDHR-HB7-IRES-GFP plasmid (generously provided by Dr. V. Planelles, University of Utah) that was digested with the same enzymes. The final bicistronic plasmid construct, pDHR-hBDNF-eGFP, was designed to co-express the hBDNF protein and the enhanced green fluorescent protein (eGFP). LVs encoding the hBDNF or control gene were generated by transient transfection of 293T cells. Vector production, concentration, and titration were performed as described previously [32],[63]–[64], except that 293T cells were used for vector titration. Briefly, 293T cells were incubated with 10-fold dilutions of concentrated virus and 8 mg/mL Polybrene (Sigma-Aldrich) for 1 hour. Fluorescence-positive cell counting to determine eGFP-positive cells was carried out at 72 hours post-transduction, and vector titers were calculated as follows: titer = *F*×*C*_0_/*V*×*D* (where *D* is the virus dilution factor, *V* is the number of 293T cells at the time of seeding per inoculum, *F* is the frequency of eGFP-positive 293T cells, and *C*_0_ is the number of 293T cells at the time of seeding per mL).

### Cell lines and culture

Human embryonic kidney (HEK) 293T cells (GenHunter Co., Nashville, TN) were maintained in Dulbecco's Modified Eagle's Medium (DMEM)(Sigma-Aldrich) containing 1.0 g/L glucose, 2 mM L-glutamine (Sigma-Aldrich), 100 IU/mL penicillin (Sigma-Aldrich), 0.1 µg/mL streptomycin (Sigma-Aldrich) and 10% fetal bovine serum (FBS) (HyClone). Three human neuronal cell lines, neuroepithelioma cells (HTB-10, or SK-N-MC), neuroblastoma cells (HTB-11 or SK-N-SH, and SH-SY5Y), and glioblastoma cells (HTB-14; or U-87) (ATCC, Manassas, VA), were cultured in Minimum Essential Medium (Eagle) (MEM) (Sigma-Aldrich) supplemented with 2 mM L-glutamine, 1.0 mM sodium pyruvate, 100 IU/mL penicillin, 0.1 µg/mL streptomycin and 10% FBS. The human embryonic microglial cell line CHME-5 (provided by Dr. Pierre Talbot, Universite du Quebec) was cultured in Dulbecco's Modified Eagle's Medium (DMEM) (Sigma-Aldrich) containing 4.5 g/L glucose, 2 mM L-glutamine, 100 IU/mL penicillin, 0.1 µg/mL streptomycin and 10% FBS. Human monocytic cell line U937 (ATCC Manassas, VA), was cultured in RPMI1640 (Sigma- Aldrich) supplemented with 2 mM L-glutamine, 1.0 mM sodium pyruvate, 100 IU/mL penicillin, 0.1 µg/mL streptomycin and 10% FBS.

### In vitro isolation and cultivation of human peripheral mononocyte-derived macrophages (hMDM) and murine myeloid monocyte-derived macrophages (mMDM)

Blood was drawn from consented healthy donors into BD Vacutainer™ ACD (Beckton Dickinson), and peripheral blood mononuclear cells (PBMC) were isolated by density-gradient centrifugation through Ficoll-Paque™ PLUS (GE Healthcare). The purified PBMC were then resuspended in RPMI-1640 medium supplemented with 10% heat-inactivated human serum (Sigma- Aldrich), 20% heat-inactivated FBS, 100 IU/mL penicillin, 100 µg/mL streptomycin sulfate and 0.292 mg/mL L-glutamine. The purified PBMC were then seeded into 25 cm^2^ tissue culture flasks (Corning) at 1.8×10^7^ cells and incubated at 37°C with 5% CO_2_ to allow the attachment of freshly isolated monocytes. Following a 3 hours incubation, non-adherent cells were removed by aspiration and the remaining adherent cells were washed extensively with Dulbecco's phosphate-buffered saline (DPBS) (Sigma), prior to the addition of 4 mL RPMI-1640 growth medium per flask, and subsequent cultivation at 37°C. Cell growth and monolayer formation were observed daily using a phase-contrast inverted microscope (Nikon).

To verify the purity of the attached cells, these monolayer cultures were stained with human CD11b monoclonal antibody conjugated with PE (Caltag Laboratories, CA, USA). Briefly, the antibody was diluted 100 times with 1% BSA (Sigma) in DPBS and hMDM cultures, and at day 7 were stained with the diluted antibody. After 1–2 hours incubation, stained cells were rinsed twice with DPBS, and then examined under an inverted fluorescence microscope.

For murine myeloid monocyte-derived macrophages (mMDM), the method was conducted according to Dou et al [30]. In brief, BALB/c mice (Frederick National Laboratory of the National Cancer Institute) were euthanized via CO_2_ chamber and femurs of the mice were removed. After removing the muscle, bone marrow cells were flushed with DPBS from the bone shafts into single-cell suspensions, and cultured for 5 days supplemented with 1000 U/mL M-CSF (Wyeth). Cultured mMDM proved to be >98% CD11b+ by immunofluorescent staining with PE-conjugated rat monoclonal antibodies directed against mouse CD11b (Caltag Laboratories, CA, USA).

### Transduction of human cell lines and primary cells

Briefly, 5×10^5^ HTB-10, HTB-11 (neuronal) or CHME-5 (microglial) cells were subcultured in 25 cm^2^ tissue culture flasks for 24 hours before transduction. For transduction, cell culture media were removed and cells were washed twice with DPBS followed by adding 0.5 mL vector suspension (1×10^7^ IU/mL, MOI = 10, for HTB-11, MOI = 1) containing 8 µg/mL polybrene, and affected cells were incubated at 37°C for 2 hours. Vector suspension was aspirated and transduced cells were added to 4 mL/flask of fresh growth medium and incubated at 37°C with 5% CO_2_. The medium was replaced 24 hours post-infection and transduction efficiencies were evaluated on day 3 post-infection. The percentage of GFP positive cells was determined by calculating the number of GFP positive cells and total cells from randomly selected microscopic fields under a fluorescence microscope (Nikon Eclipse TE2000-U). A total of 5 microscopic fields, each containing at least 100 cells, were counted for each transduction test.

hMDM were seeded to 12.5 cm^2^ tissue culture flasks at a density of 1.8×10^7^ cells per flask. Six days later, transductions were performed using MOI = 50 by thawing the titrated virus stocks at room temperature, mixing the appropriate volume of virus concentrate with 8 mg/mL Polybrene (Sigma), and adding the mixture to the target cells together with RPMI to achieve a total volume of 1 mL per flask. mMDM were seeded to 12.5 cm^2^ tissue culture flasks at a density of 1.0×10^7^ cells per flask. Four days later, transductions were performed using MOI = 50 by thawing the titrated virus stocks at room temperature, mixing the appropriate volume of virus concentrate with 8 mg/mL Polybrene (Sigma), and adding the mixture to the target cells together with RPMI to achieve a total volume of 1 mL per flask. After 2-hour incubation at 37°C, an additional 3 mL of complete RPMI was added per flask. Most of the culture medium was aspirated and replaced by fresh RPMI in the day following transduction. The transduced cells were examined daily using visual inspection by inverted light and fluorescence microscopy.

### Enzyme-linked immunosorbent assay (ELISA)

A 96-well plate was coated with rabbit polyclonal antibodies to human hBDNF (Antigenix America, USA) and incubated overnight at 4°C. The plate was washed three times with 0.05% Tween-20 in PBS and blocked with 1% BSA (Sigma-Aldrich) for 1 hour at room temperature on an orbital shaker. After washing three times with PBS, the plate was incubated with diluted hBDNF containing supernatant samples for 1 hour and then incubated with biotin-conjugated rabbit polyclonal antibodies to human hBDNF (Antigenix America, USA) for 1 hour. The plate was then washed and incubated with streptavidin-horseradish peroxidase (Rockland) for 1 hour at room temperature. The presence of human hBDNF protein was detected with one-Step Ultra TMB (tetramethylbenzidine) (Pierce). The enzymatic reaction was stopped via the addition of 1 M sulfuric acid. The quantitation of hBDNF protein was based on optical density values at 450 nm with 570 nm as the reference wavelength, and compared with a standard curve of purified human hBDNF protein (Antigenix America, USA), using an ELISA reader (Beckman Coulter AD340).

### Western blot

The supernatant or lysate of transduced- or non-transduced- cells including HTB-10, HTB-11, and CHME-5 cells, was mixed with 6× sodium dodecyl sulfate (SDS) sample buffer (100 mM Tris-HCl at pH 6.8, 200 mM dithiothreitol, 4% SDS, 0.2% bromophenol blue, and 20% glycerol) and loaded on 5% stacking/15% separating SDS-polyacrylamide gels. Following electrophoresis at 30 mA for 1 hour, separated proteins were transferred onto a nitrocellulose membrane (NCM) (Invitrogen, Carlsbad, CA). The membranes were saturated with 1% bovine serum albumin (BSA) (Sigma-Aldrich) in TBST buffer containing 10 mM Tris- HCl at pH 8.0, 150 mM NaCl, and 0.05% Tween-20 for 1 hour at room temperature, followed by overnight incubation with diluted rabbit polyclonal antibodies to human hBDNF (Antigenix America, USA) at 4°C. Following extensive washing with TBST, the NCM was incubated with diluted biotin-conjugated rabbit polyclonal antibodies to human hBDNF (Antigenix America, USA) at room temperature for 1 hour, then washed three times with TBST and exposed to a 3,3-diaminobenzidine tetrahydrochloride (DAB) substrate (PIERCE, Rockford, IL) for identification of protein bands.

### MTT assay

Cells at the exponential growth phase were harvested using trypsin-versene solution (Sigma) and seeded into 96-well plates at 2×10^4^ cells/well in 100 µL. Following overnight incubation and the formation of a cell monolayer, test cells were treated with Tumor necrosis factor-1 alpha (TNF-α) at selected concentrations. Seventy-two hours later, 20 µL of MTT solution (5 mg/mL) was added to the 100 µL of medium in each well, and the plate incubated at selected temperatures for 4 hour. The solution was removed followed by adding 100 µL/well of DMSO to solubilize the purple formazan crystals produced. Absorbance in each well was measured at 570 nm using a 96-well plate reader (Beckman Coulter, Fullerton, CA).

### Immunochemistry

Expression of eGFP was evaluated by immunochemical staining using selected monoclonal antibodies conjugated with TRITC. Briefly, cells in 48-well plates were washed twice with DPBS, then blocked by DPBS containing 0.5% (w/v) bovine serum albumin (BSA), and then directly stained with the appropriately diluted primary antibody - goat anti-green fluorescent protein (GFP) antibody (Rockland, USA). After incubation for 2 hours in a dark room, the cells were washed with DPBS. The secondary antibody used was Donkey anti-Goat IgG with TRITC (Rockland, USA).Cells were visually examined and documented using an inverted fluorescence microscope (Nikon Eclipse TE2000-U) with a digital camera attachment.

### Neuroprotection assay

A neuroprotection assay was conducted to assess the degree of that hBDNF in neutralizedthe neurotoxic effects of TNF-α (R & D, USA). LV-hBDNF-HTB-10 cells, mock cells, and non-transduced HTB-10 cells were all exposed to TNF-α at three different concentrations: 500 ng/mL, 100 ng/mL, and 20 ng/mL, respectively. After 48 hours, MTT assay was performed to determine cell viability by absorbance reading at 570 nm using a microplate reader (Beckman Coulter AD340). T-tests were used to assess any significant differences in cell viability between transduced and non-transduced cells as well as cells receiving conditioned media from transduced or non-transduced cells.

Similarly, HTB-10 cells were exposed to TNF-α at 300 ng/mL, 150 ng/mL, and 75 ng/mL, diluted with culture medium or conditioned culture medium (1∶10 dilution) collected from LV-hBDNF-transduced cells. The cell viability in normal culture media or conditioned culture media without TNF-α is normalized to be 100%. Additionally, U937 cells were exposed to 16 ng/mL, 8 ng/mL, 4 ng/mL, 2 ng/mL and 1 ng/mL TNF-α, which was diluted with culture medium or conditioned culture media from LV-hBDNF-transduced cells. The cell viability from HTB-10 cells not exposed to TNF-α, but normal medium or conditioned medium (1∶10 dilution of collected medium from LV-hBDNF-transduced cells) alone was considered 100%. After 4-hour incubation, MTT assay was performed to determine cell viability with results read at 570 nm using a microplate reader (Beckman Coulter AD340).

SH-SY5Y cells (ATCC, Manassas, VA) were pretreated with conditioned media (1∶10 & 1∶20 dilutions) from LV-hBDNF-transduced HTB-11 cells for 1 h, then exposed to HIV-1 Tat protein (200 ng/mL) for 48 hours, and cell viability was determined by MTT assay as described above. T-tests were used to assess any significant differences in cell viability between cells receiving conditioned media from transduced or non-transduced cells.

### GeXP (Genetic analysis system)

Transduced primary hMDM were analyzed at day 6 post-transduction. The expression of 24 reference genes was analyzed using GenomeLab GeXP Genetic Analysis System (Beckman Coulter, Brea, CA). RNA was extracted from hMDM using Qiagen RNeasy Kit (cat# 74104). First, cells were scraped and pelleted through centrifugation at 4,000 RPM for 5 min. The supernatant was discarded and the cell pellets were resuspended in resuspension buffer. The cells were lysed with lysis buffer and neutralized with neutralization buffer. Cell debris was removed and RNA was captured using an RNeasy spin column, washed twice with wash buffer, and then eluted with RNase free molecular grade water. RNA concentration was determined via spectrophotometer at 260 nm. Human Reference RNA (Beckman Coulter, A54267) was used to develop a standard curve for a human reference multiplex. XP-PCR was performed on sample RNA with reverse transcriptase minus and the test included no template reactions as negative controls and reference RNA as a positive control. Kan^R^ RNA (Beckman Coulter, #A85017) was added to each sample as an internal control and two genes were designated as reference genes. XP-PCR was conducted in two stages: the first was the RT reaction in which sample RNA was incubated with DNase/RNase free water, 5× RT buffer, reverse transcriptase, and the reverse primer plex for 1 min at 48°C, 60 min at 42°C, and 5 min at 95°C. And next, PCR was performed where cDNA from the RT reaction was incubated with 5× PCR buffer, 25 mM MgCl_2_, DNA polymerase, and the forward primer plex for 10 min at 95°C, and 35 cycles of 30 s at 94°C, 30 s at 55°C, and 1 min at 70°C. Finally, amplicons were combined with DNA size standard-400 and sample loading solution (Beckman Coulter, #608098 and #608082), then subjected to capillary electrophoresis. The results were analyzed by Express Profiler and Quant Tool (Beckman Coulter)and normalized to the internal control Kan^R^ to eliminate any inter-capillary differences and then normalized a second time to the pre-selected reference genes. Finally, results were compared to the standard curve created for the specific multiplex and normalized values and significance differences between samples were calculated using T-tests.

### Statistical analysis

T-tests were employed in this study for statistical analysis (using Prism software). * indicates 0.01<*p*≤0.05; ** indicates *p*≤0.01. The data set was exported from GeXP software after normalization to kanamycin. All multiplexes were combined into one Excel data file. The normalized intensity of each replicate was used to calculate an average intensity for each group (i.e. transduced cells and non-transduced cells). The sample groups were compared to each other using a two-tailed T-test, with statistical significance set at *p*<0.05 (n = 4). Normalized values were displayed in a bar graph using Excel.

## Conclusions

Our study demonstrated that moderate to high levels of hBDNF transduction efficiency and expression can be obtained through the use of lentiviral vector system in human neuronal cell lines as well as primary cultures of human MDM. Moreover, the hBDNF produced from transduced cells is able to provide significant protection against TNF-α and HIV-1 Tat-mediated neurotoxicity. These in vitro study findings further research support that utilize genetically modified monocytes/macrophages as a possible gene delivery vehicle into the brain, with cell-mediated expression of hBDNF as a novel therapy for neuroAIDS.
